# Concurrent Eclampsia, HELLP (Hemolysis, Elevated Liver Enzymes, and Low Platelets) Syndrome and Peripartum Cardiomyopathy in a Primigravida: A Case Report

**DOI:** 10.7759/cureus.106796

**Published:** 2026-04-10

**Authors:** Rekha Anbu, Pravin SJ, Vineil Cruz, Yasodha S, Esha Yenugonda, Akhil Ashirwadh

**Affiliations:** 1 Obstetrics and Gynaecology, Bhaarath Medical College and Hospital, Chennai, IND; 2 Medicine and Surgery, Bhaarath Medical College and Hospital, Chennai, IND; 3 Medicine, Bhaarath Medical College and Hospital, Chennai, IND

**Keywords:** acute cardiogenic pulmonary edema, case report, eclampsia, hellp syndrome, hypertensive disorders of pregnancy, peripartum cardiomyopathy, point-of-care-ultrasound

## Abstract

Hypertensive disorders of pregnancy can rapidly progress to multi-organ failure. Eclampsia and HELLP (hemolysis, elevated liver enzymes, and low platelet count) syndrome are obstetric emergencies, while peripartum cardiomyopathy (PPCM) is a rare but potentially fatal cause of heart failure in late pregnancy and the postpartum period. We report the case of a critically ill 32-year-old primigravida at 35 weeks’ gestation with treated hypothyroidism who presented with generalized tonic-clonic seizures, severe hypertension, and pulmonary edema.

Laboratory evaluation demonstrated thrombocytopenia and transaminitis with markedly elevated lactate dehydrogenase (LDH), consistent with HELLP syndrome. Obstetric assessment revealed a closed cervical os with the vertex above the brim, and bedside ultrasound confirmed a viable fetus with good cardiac activity and no evidence of placental abruption. Targeted echocardiography showed global left ventricular hypokinesia with severe systolic dysfunction (estimated ejection fraction ~25%) and a dilated left ventricle with moderate (grade II) mitral regurgitation and diastolic dysfunction.

After stabilization with airway protection, magnesium sulfate, antihypertensive therapy, diuretics, and inotropic support, an emergency lower-segment cesarean section was performed, resulting in the delivery of a live female baby. Postoperatively, the patient required intensive care, blood product transfusion for HELLP syndrome, and guideline-directed heart failure therapy. Serial echocardiography showed improvement in ejection fraction to 45% on postoperative day two and 58% before discharge. This report underscores the diagnostic overlap between preeclampsia-spectrum pulmonary edema and PPCM, and highlights the role of bedside echocardiography and multidisciplinary critical care in optimizing maternal outcomes.

## Introduction

Eclampsia (defined as new onset generalized seizures in a patient with severe preeclampsia) remains an obstetric emergency associated with significant maternal and perinatal morbidity and mortality [1]. HELLP (hemolysis, elevated liver enzymes, and low platelet count) syndrome represents a severe form of preeclampsia-spectrum disease and is associated with potentially life-threatening maternal and fetal complications [2]. Peripartum cardiomyopathy (PPCM) is an idiopathic cardiomyopathy that presents as heart failure due to left ventricular systolic dysfunction (left ventricular ejection fraction (LVEF) <45%) in the peripartum period, after excluding other causes [3]. Hypertensive disorders of pregnancy, including preeclampsia and HELLP, are consistently linked to PPCM; a systematic review and meta-analysis demonstrated a strong association between preeclampsia and PPCM [4].

Severe hypertensive disease can present with acute pulmonary edema and hemodynamic instability that clinically resembles PPCM, making early cardiac assessment essential. In real-world practice, distinguishing these entities is particularly challenging because both conditions share overlapping features, including respiratory distress, reduced oxygen saturation, and radiographic pulmonary congestion, while the hemodynamic profiles and treatment priorities differ substantially. Furthermore, when all three conditions occur simultaneously, the absence of a preceding cardiac history and the acuity of the obstetric emergency can delay the recognition of an underlying cardiomyopathy.

A recent case report in Cureus highlights the role of focused bedside echocardiography in differentiating hypertensive pulmonary edema from PPCM and guiding condition-specific management [5]. Recent state-of-the-art reviews emphasize that PPCM symptoms may closely resemble those of normal pregnancy, that echocardiography is essential for diagnosis, and treatment generally follows the standard approach for heart failure with reduced ejection fraction. Still, it is adapted to ensure safety during pregnancy and breastfeeding [6]. We present a rare case of all three conditions occurring concurrently in a primigravida, with novelty stemming from the simultaneous presentation, the diagnostic uncertainty introduced by acute pulmonary edema, and the pivotal role of early bedside echocardiography in distinguishing cardiogenic from hypertensive causes and directing multidisciplinary management.

## Case presentation

A 32-year-old primigravida at 35 weeks’ gestation with a history of treated hypothyroidism was brought to the emergency department after two episodes of generalized tonic-clonic seizures (one at home and another en route). On arrival, she was in a postictal state, agitated and noncooperative.

Examination

Initial vital signs demonstrated severe hypertension (190/120 mmHg), tachycardia (118 beats/min), and tachypnea (26 breaths/min). The patient had marked generalized edema (anasarca) with bilateral pedal edema. Respiratory examination revealed bilateral basal crepitations, raising concerns for pulmonary edema. Abdominal examination revealed a gravid uterus corresponding to 34-36 weeks’ gestation; fetal heart sounds were present. Per vaginal examination performed after initial stabilization showed an unaffected cervix with a closed os; the vertex was above the pelvic brim.

Obstetric assessment

A bedside obstetric ultrasound was performed to exclude placental abruption and confirmed an intrauterine fetus with good cardiac activity and a reassuring fetal heart rate.

Investigations

Hemoglobin was 9.98 g/dL with severe thrombocytopenia (platelet nadir 0.75×10^5^/µL). Liver enzymes were elevated (AST 196 IU/L, ALT 78 IU/L) with markedly raised lactate dehydrogenase (LDH 1770 U/L) and elevated uric acid (7.5 mg/dL), supporting HELLP syndrome. Urinalysis revealed proteinuria (2+) with hematuria (2+). Renal function was preserved (creatinine 0.9 mg/dL; urea 21 mg/dL). Serum electrolytes were as follows: Na 135 mEq/L, K 4.5 mEq/L, and Cl 108 mEq/L. Coagulation profile showed prolonged prothrombin time (PT 14.46 seconds; INR 1.08) and mildly prolonged activated partial thromboplastin time (aPTT 32.27 seconds); fibrinogen was 398.58 mg/dL. Peripheral blood smear showed mild microcytic hypochromic anemia with mild neutrophilia and moderate thrombocytopenia, with evidence of hemolysis. Serum calcium and magnesium were 8.5 mg/dL and 3.3 mg/dL, respectively (Table 1). On arrival, ABG could not be performed because the patient had a Glasgow Coma Scale (GCS) score >8 (moderate), and oxygen saturation was maintained on 5 L/min oxygen.

**Table 1:** Key laboratory investigations AST: aspartate aminotransferase; SGOT: serum glutamic-oxaloacetic transaminase; ALT: alanine aminotransferase; SGPT: serum glutamic-pyruvic transaminase; LDH: lactate dehydrogenase; PT: prothrombin time; INR: international normalized ratio; aPTT: activated partial thromboplastin time; HELLP: hemolysis, elevated liver enzymes, and low platelet count; Na: sodium; K: potassium; Cl: chloride; MgSO₄: magnesium sulfate

Test	Result	Reference range	Units	Interpretation/notes
Hemoglobin	9.98	11–14	g/dL	Anemia
Platelet count	0.75	1.5–4.0	Lakhs/cu mm	Severe thrombocytopenia
Peripheral blood smear (impression)	Mild microcytic hypochromic anemia; mild neutrophilia; moderate thrombocytopenia	Not applicable	—	Schistocytes/hemolysis seen
AST (SGOT)	196	0–35	IU/L	Elevated (HELLP)
ALT (SGPT)	78	0–49	IU/L	Elevated (HELLP)
LDH	1770	207–414	U/L	Markedly elevated (HELLP)
Uric acid	7.5	2.6–6.0	mg/dL	Elevated
Creatinine	0.9	0.6–1.1	mg/dL	Normal/near-normal
Urea	21	10–40	mg/dL	Normal/near-normal
Na	135	136–145	mEq/L	Within reference range
K	4.5	3.5–5.1	mEq/L	Within reference range
Cl	108	96–106	mEq/L	Mildly elevated
PT	14.46	11–15		Prolonged
INR	1.08	0.9–1.20	—	Within reference range / mildly elevated
aPTT	32.27	30–40	Seconds	Mild prolongation
Fibrinogen	398.58	200–400	mg/dL	Within reference range
Serum calcium	8.5	8.8–11.2	mg/dL	Low
Serum magnesium	3.3	1.77–2.58	mg/dL	Elevated/therapeutic (on MgSO₄)

Given the combination of seizures, severe hypertension, and laboratory derangements, the working diagnosis was eclampsia complicated by HELLP syndrome.

Echocardiography

**Figure 1:**
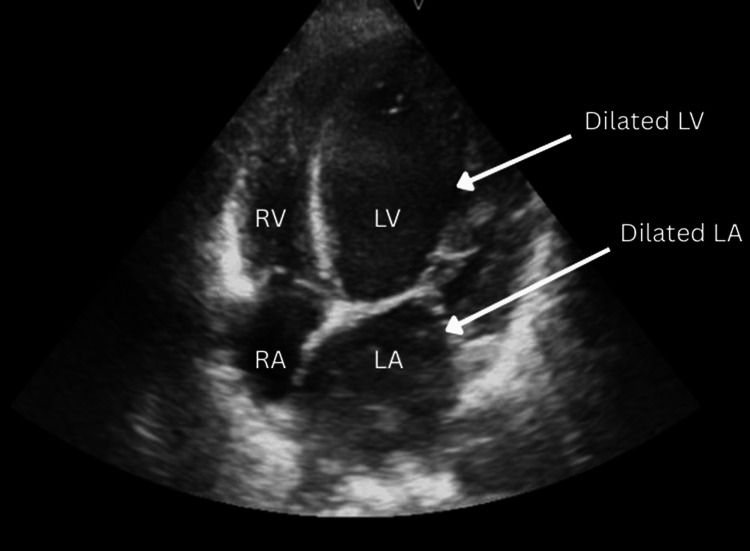
Transthoracic echocardiography (apical four-chamber view) - image 1 Two-dimensional image demonstrating global left ventricular hypokinesia with severe left ventricular grade II diastolic dysfunction and dilated left ventricle with grade II mitral regurgitation; mild (grade I) tricuspid regurgitation was also noted RV: right ventricle; RA: right atrium; LV: left ventricle; LA: left atrium

**Figure 2:**
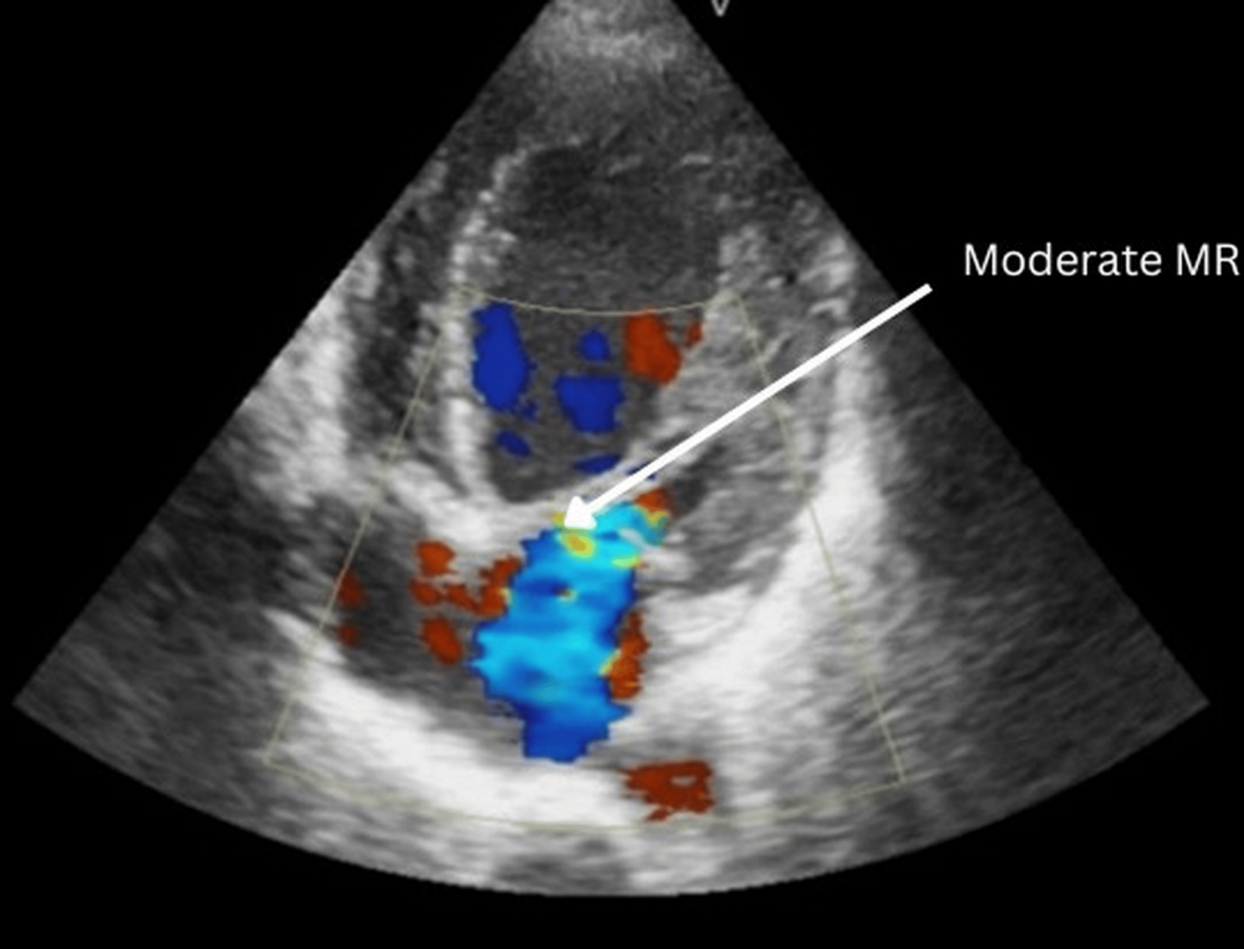
Transthoracic echocardiography (apical four-chamber view) - image 2 Color Doppler image demonstrating a dilated left ventricle with grade II mitral regurgitation. Chamber labels (RV, RA, LV, and LA) are provided; arrows highlight the dilated LV/LA and the moderate mitral regurgitation jet RV: right ventricle; RA: right atrium; LV: left ventricle; LA: left atrium

Apical four-chamber view demonstrated global left ventricular hypokinesia with severe systolic dysfunction (visually estimated ejection fraction ~25%), a dilated left ventricle, grade II diastolic dysfunction, grade II mitral regurgitation, and mild (grade I) tricuspid regurgitation (Figure 1). Color Doppler apical four-chamber view confirmed a moderate (grade II) mitral regurgitation jet (Figure 2).

Chest X-ray

**Figure 3:**
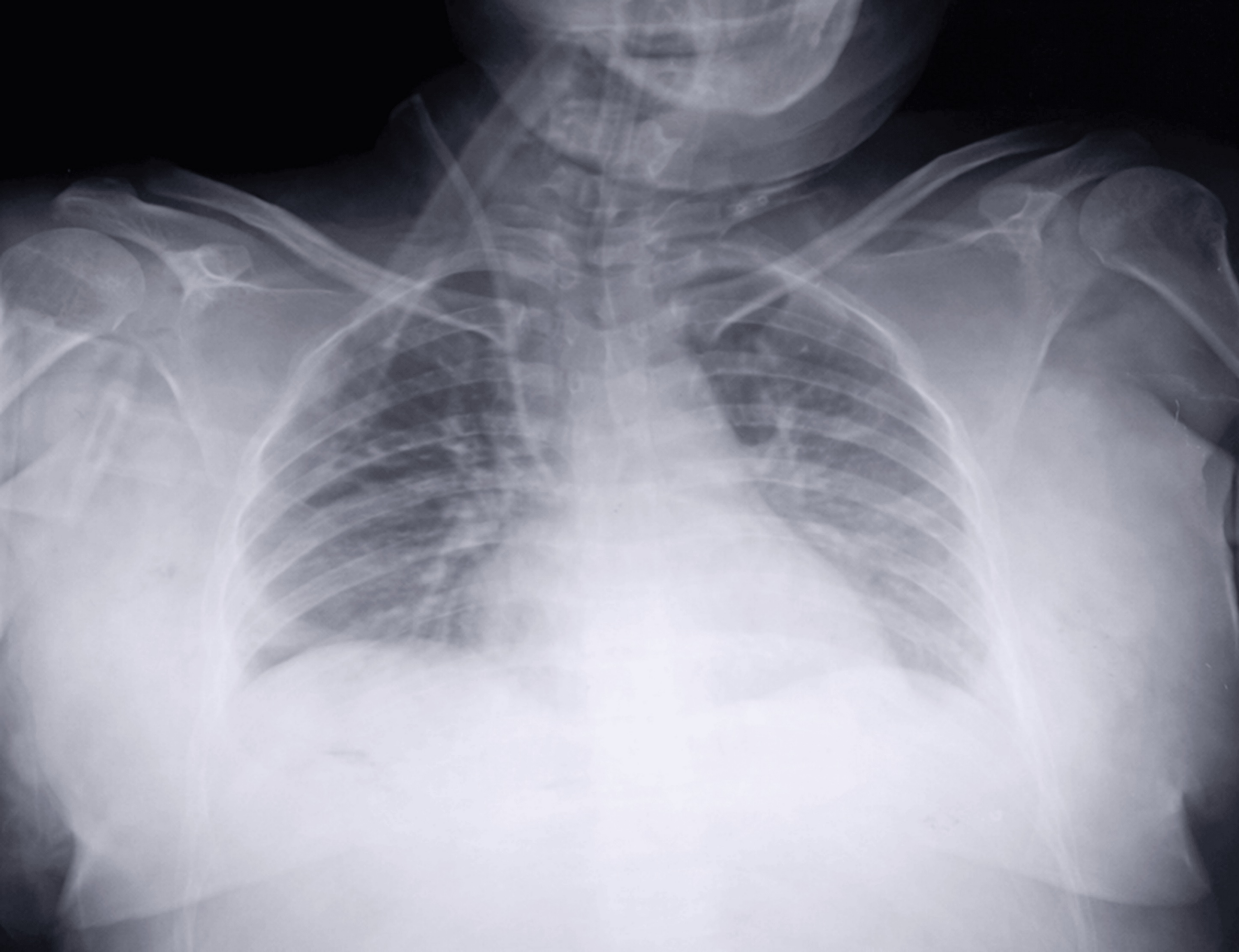
Chest radiograph taken shortly after ICU admission Although cardiomegaly is ideally assessed on a posteroanterior view, this portable anteroposterior film suggests an enlarged cardiac silhouette (estimated cardiothoracic ratio >0.5) with bilateral lower-zone air-space opacities consistent with pulmonary edema ICU: intensive care unit

A portable anteroposterior chest radiograph obtained during the acute presentation showed an enlarged cardiac silhouette with bilateral lower-zone air-space opacities consistent with pulmonary edema (Figure 3).

Management and outcome

The patient was managed as a critical obstetric emergency. Airway protection and mechanical ventilation were instituted due to depressed mental status. She received intravenous benzodiazepine (lorazepam 2mg IV stat) and Inj levetiracetam 1 g IV stat, followed by 500 mg BD for acute seizure control, followed by magnesium sulfate loading and maintenance infusion for eclampsia management, along with IV labetalol 20 mg bolus followed by 40 mg for blood pressure control. Given the high-risk maternal condition with suspected HELLP syndrome and PPCM, an emergency lower-segment cesarean section was performed under general anesthesia. A live female neonate weighing 1.895 kg was delivered with Apgar scores of 8 and 9 at one and five minutes, respectively. Peri-intubation and induction vital parameters are summarized in Table 2.

**Table 2:** Vital parameters at key time points during airway management and perioperative stabilization ICU: intensive care unit

Time point	Blood pressure (mmHg)	Heart rate (bpm)	Saturation (SpO₂)
Arrival	190/120	118	82%
After intubation	150/100	97	97–99%
At induction	180/100	101	100%
After induction	140/80	84	100%
Before transfer to the ICU	140/90	89	100%

Postoperatively, the patient required ICU management with ongoing ventilatory support, diuresis, and afterload reduction (intravenous furosemide and nitroglycerin infusion) and inotropic support (dobutamine) for PPCM-related pulmonary edema. She received blood products (packed red blood cells, fresh frozen plasma, and platelets) for HELLP-associated cytopenias and coagulopathy. Broad-spectrum antibiotics (Inj Piptaz 4.5g IV TDS) were administered, and thromboprophylaxis with low molecular weight heparin was initiated after stabilization.

Magnesium sulfate was continued for 24 hours postoperatively. ABG was not obtained at presentation as noted above; the first postoperative ABG (ABG-1) is summarized in Table 3, and a subsequent ABG (ABG-2) later during ICU stay is summarized in Table 4.

**Table 3:** Arterial blood gas parameters immediately after surgery (ABG-1)

Parameter	Result	Units
pH	7.30	—
pCO₂	34.7	mmHg
pO₂	190	mmHg
HCO₃⁻	16.6	mmol/L

**Table 4:** Arterial blood gas parameters later during ICU stay (ABG-2) ICU: intensive care unit

Parameter	Result	Units
pH	7.41	—
pCO₂	36.3	mmHg
pO₂	62	mmHg
HCO₃⁻	22.4	mmol/L

The patient was extubated on postoperative day one. Serial echocardiography demonstrated improvement in left ventricular function (ejection fraction 45% on postoperative day two and 58% before discharge) (Table 5). She was transitioned to oral antihypertensive and heart failure medications, including enalapril and carvedilol, and discharged in stable condition with advice for close outpatient follow-up and repeat echocardiography.

**Table 5:** Echocardiography trend during hospitalization LVEF: left ventricular ejection fraction; LV: left ventricular; EF: ejection fraction; MR: mitral regurgitation; TR: tricuspid regurgitation

Time point	Key findings	LVEF
Preoperative (bedside echo)	Global LV hypokinesia; dilated LV; grade II mitral regurgitation; grade II diastolic dysfunction	~25%
Postoperative day 2	EF has improved	45%
Before discharge	Trivial MR; trivial TR; no pericardial effusion	58%

## Discussion

Hypertensive disorders of pregnancy can worsen quickly and require immediate maternal stabilization with seizure management, treatment of sudden severe hypertension, and timely delivery when indicated [1]. HELLP syndrome adds substantial maternal risk due to hemolysis, hepatic injury, thrombocytopenia, and potential progression to serious complications that affect perioperative planning and transfusion strategy [2]. PPCM is defined by the new onset of heart failure with left ventricular systolic dysfunction (LVEF <45%) late in pregnancy or in the months following delivery, after exclusion of alternative causes, and echocardiography is essential for both diagnosis and monitoring [3]. A systematic review and meta-analysis demonstrate a strong association between preeclampsia and PPCM, supporting a low threshold for cardiac evaluation when respiratory compromise occurs in preeclampsia spectrum disease [4].

Because hypertensive pulmonary edema and decompensated PPCM can present with overlapping clinical findings, targeted bedside echocardiography can help distinguish the underlying physiology and guide fluid and vasoactive decisions in time-critical situations [5]. Contemporary state-of-the-art reviews emphasize early recognition of PPCM and initiation of appropriate postpartum heart failure therapy when clinically feasible, with follow-up to document recovery [6]. Comprehensive genetic and clinical evidence highlights that peripartum cardiomyopathy spans a spectrum ranging from genetic factors to clinical management, and ESC guidelines on cardiovascular diseases during pregnancy provide structured frameworks for diagnosis and treatment in this population [7,8]. Prognosis and counseling are closely linked to left ventricular recovery, and future pregnancy risk is particularly important when left ventricular function fails to normalize [9].

Management is optimized when care is coordinated through a multidisciplinary cardio-obstetric approach involving obstetrics, anesthesia/critical care, and cardiology, particularly in complex unstable presentations [9]. Guideline-based treatment of pregnancy-related severe hypertension prioritizes prompt antihypertensive therapy to reduce maternal stroke and end-organ injury, with commonly used first-line agents including intravenous labetalol or hydralazine and oral immediate-release nifedipine based on clinical context and availability [9]. Chinese clinical practice guidance similarly endorses labetalol and nifedipine as commonly used agents and provides practical dosing approaches for hypertension and preeclampsia in pregnancy [10].

Focused cardiac ultrasound/point-of-care echocardiography is supported by professional consensus as a rapid, repeatable bedside method to evaluate ventricular function and major valvular pathology in acute care settings [11]. When HELLP-associated thrombocytopenia is present, an interdisciplinary obstetric anesthesia consensus supports individualized neuraxial decision-making and generally considers neuraxial procedures reasonable at platelet counts ≥70,000/µL in the absence of additional bleeding risk factors [12]. After delivery, guideline-directed therapy for heart failure with reduced ejection fraction should be initiated and titrated as tolerated, with attention to blood pressure, renal function, and breastfeeding considerations [13]. American heart failure guidelines similarly recommend foundational HFrEF therapies with ongoing follow-up and repeat imaging to guide long-term management and confirm recovery [14]. Chinese expert consensus on PPCM also emphasizes structured follow-up, repeat echocardiography, and counseling regarding recurrence risk and future pregnancy planning [15].

In our patient, early bedside echocardiography enabled prompt detection of severe systolic dysfunction and supported timely hemodynamic optimization, with serial imaging documenting recovery and supporting safe transition to oral therapy [3,5,11]. Neurological status was assessed using the Glasgow Coma Scale [16], and neonatal condition was evaluated using the Apgar scoring system [17], both of which are standardized assessment tools integral to emergency obstetric and neonatal care.

## Conclusions

The simultaneous occurrence of eclampsia, HELLP syndrome, and peripartum cardiomyopathy represents a high-risk and time-critical obstetric emergency. Because pulmonary edema due to severe preeclampsia can closely resemble PPCM, early targeted echocardiography should be considered when respiratory compromise is present, as the diagnosis directly influences anesthetic planning, fluid strategy, and the need for inotropic or condition-specific heart failure therapy. Prompt stabilization, expedited delivery, and multidisciplinary ICU care contributed to favorable outcomes for both mother and neonate in this case.
